# Nasal biomarkers of immune function differ based on smoking and respiratory disease status

**DOI:** 10.14814/phy2.15528

**Published:** 2023-02-13

**Authors:** Meghan E. Rebuli, Anna Stanley Lee, Lina Nurhussien, Usman A. Tahir, Wendy Y. Sun, Adam J. Kimple, Charles S. Ebert, Martha Almond, Ilona Jaspers, Mary B. Rice

**Affiliations:** ^1^ Department of Pediatrics and Curriculum in Toxicology and Environmental Medicine University of North Carolina at Chapel Hill Chapel Hill North Carolina USA; ^2^ Center for Environmental Medicine, Asthma and Lung Biology University of North Carolina at Chapel Hill Chapel Hill North Carolina USA; ^3^ Department of Medicine, Beth Israel Deaconess Medical Center Harvard Medical School Boston Massachusetts USA; ^4^ Department of Otolaryngology‐Head and Neck Surgery University of North Carolina at Chapel Hill Chapel Hill North Carolina USA

**Keywords:** cigarette smoking, COPD, CRS, immune mediators, nasal mucosa

## Abstract

Respiratory biomarkers have the potential to identify airway injury by revealing inflammatory processes within the respiratory tract. Currently, there are no respiratory biomarkers suitable for clinical use to identify patients that warrant further diagnostic work‐up, counseling, and treatment for toxic inhalant exposures or chronic airway disease. Using a novel, noninvasive method of sampling the nasal epithelial lining fluid, we aimed to investigate if nasal biomarker patterns could distinguish healthy nonsmoking adults from active smokers and those with chronic upper and lower airway disease in this exploratory study. We compared 28 immune mediators from healthy nonsmoking adults (*n* = 32), former smokers with COPD (*n* = 22), chronic rhinosinusitis (CRS) (*n* = 22), and smoking adults without airway disease (*n* = 13). Using ANOVA, multinomial logistic regressions, and weighted gene co‐expression network analysis (WGCNA), we determined associations between immune mediators and each cohort. Six mediators (IL‐7, IL‐10, IL‐13, IL‐12p70, IL‐15, and MCP‐1) were lower among disease groups compared to healthy controls. Participants with lower levels of IL‐10, IL‐12p70, IL‐13, and MCP‐1 in the nasal fluid had a higher odds of being in the COPD or CRS group. The cluster analysis identified groups of mediators that correlated with disease status. Specifically, the cluster of IL‐10, IL‐12p70, and IL‐13, was positively correlated with healthy and negatively correlated with COPD groups, and two clusters were correlated with active smoking. In this exploratory study, we preliminarily identified groups of nasal mucosal mediators that differed by airway disease and smoking status. Future prospective, age‐matched studies that control for medication use are needed to validate these patterns and determine if nasosorption has diagnostic utility for upper and lower airway disease or injury.

## Graphical abstract

Respiratory biomarkers have the potential to identify airway injury by revealing inflammatory processes within the respiratory tract. Using a novel, noninvasive method of sampling the nasal epithelial lining fluid, we aimed to investigate if nasal biomarker patterns could distinguish healthy nonsmoking adults from active smokers and those with chronic upper and lower airway disease. In this exploratory study, we identified groups of nasal mucosal mediators that differed by airway disease and smoking status.

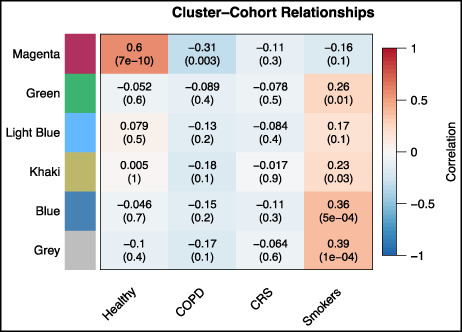

## 1 INTRODUCTION

Chronic upper and lower airway disease, including chronic rhinosinusitis (CRS) and chronic obstructive pulmonary disease (COPD), require long‐term management and frequent medical care due to symptom exacerbation (Criner & Han, 2018; Denneny et al., 2019; Kumbhare et al., 2016; Tan et al., 2013). Both conditions are caused or exacerbated by toxic inhalant exposures, especially tobacco smoke (Reh et al., 2012). Currently, there are neither respiratory biomarkers to evaluate airway injury nor to help clinicians determine which patients might warrant further diagnostic work‐up, counseling, and treatment for toxic inhalant exposures or chronic airway disease.

Efforts in precision medicine to develop disease biomarkers may help address this need. An extensive body of research has evaluated serum biomarkers of systemic inflammation and their associations with lung function and respiratory disease (Jain, 2017; Stockley et al., 2019). Some biomarkers appear to have some diagnostic utility for COPD when elevated in the bloodstream, although negative studies contradicting these findings have also emerged (Celli et al., 2019). Such findings on serum biomarkers may not translate to the inflammatory processes within the respiratory tract of patients with chronic airway disease or smoking‐related injury (Gianniou et al., 2016; Hurst et al., 2005; Rebuli et al., 2018). We have previously demonstrated that levels of immune mediators measured in upper respiratory compartment, particularly the nose, and lower respiratory compartments (e.g., sputum), correlate well with each other, but poorly with levels of the same mediators measured in the bloodstream (Payton et al., 2022). We posit that the upper airway, including the nose, which shares physiology and histomorphology with the lower airway according to the “unified airway” hypothesis, is a more relevant sampling site for respiratory biomarker research (Chang, 2014; Giavina‐Bianchi et al., 2016). The upper airway is convenient for quick and noninvasive sample collection via the nose, which alleviates cost, effort, and patient burden to obtain clinical or research samples. In contrast, lower airway samples (e.g., bronchoalveolar lavage, induced sputum) are time consuming and more invasive to collect, which may deter investigators and study participants from collecting this type of sample, especially for large‐scale observational or clinical studies. Members of our research team have developed a novel, minimally invasive method for sampling the nasal epithelial lining fluid as an indicator of immune activity within the upper respiratory mucosal environment (Rebuli et al., 2017).

In this multicenter exploratory, cross‐sectional study, we applied this nasal sampling methodology to assess if levels of immune mediators in the nasal fluid differ among adults with moderate‐to‐severe COPD, CRS, and active tobacco smoking compared to healthy nonsmoking adults. We hypothesized that clusters of nasal biomarkers would help distinguish healthy adults from those with chronic upper and lower airway disease and active tobacco use. Ultimately, evaluation of the nasal epithelial lining fluid may permit evaluation of biologically relevant immune responses to environmental exposures, treatments, and interventions for chronic airway disease.

## 2 METHODS

### 2.1 Participant recruitment

Nasal epithelial lining fluid (NELF) was collected from four small cohorts of individuals: adults with COPD, CRS, active cigarette smokers, and healthy controls to complete an exploratory analysis of nasal immune mediator production across groups. Participant recruitment and sample collection protocols were approved by the University of North Carolina at Chapel Hill Institutional Review Board (for the CRS, active smoker, and healthy control cohorts) and Beth Israel Deaconess Medical Center Institutional Review Board (for the COPD cohort).

Inclusion criteria for the COPD cohort (*N* = 22) included: former smokers with at least a 10 pack‐year smoking history, clinical diagnosis of COPD, and at least moderate GOLD Stage II airflow obstruction, defined as forced expiratory volume in 1 s (FEV1)/forced vital capacity (FVC) ratio of <70% and FEV1 < 80% predicted. Exclusion criteria for the COPD cohort was active smoking. Participants with a history of lung cancer, interstitial lung disease, or bronchiectasis were ineligible to participate. Key inclusion criteria for CRS cohort (*N* = 22) included: physician diagnosis of CRS with plans for surgical treatment, while exclusion criteria were diagnosis of cystic fibrosis, vasculitis, nasal tumor, or ongoing immunosuppressant therapy. CRS NELF samples were collected prior to surgical treatment. Key inclusion criteria for the cigarette smoking cohort (*N* = 13) included current use of five or more cigarettes per day on average, forced expiratory volume in 1 s (FEV1) greater than 75% of predicted, no current symptoms of allergic rhinitis, no diagnosis or symptoms of asthma, COPD, cardiac disease, bleeding disorders, or immunodeficiency, and no recent nasal surgery or nasal steroid use. Key inclusion and exclusion criteria for the healthy controls (*N* = 32) were the same as the cigarette smoker cohort, except that they had no history of tobacco product use. The healthy control group was split into two sub‐cohorts for initial analyses and the combined overall cohort results are reported here as there were no significant differences in the sub‐cohort analyses. Smoking status was accounted for in each group and is reported in Table 1.

**TABLE 1:** Participant characteristics by cohort

	Nonsmokers (healthy)	COPD	CRS	Smokers	Total
	*N* = 32	*N* = 22	*N* = 22	*N* = 13	*N* = 89
Age (*SD*)	29.0 (7.6)	70.9 (8.0)****	41.3 (13.5)****	31.8 (6.1)	42.8 (19.2)
Gender (% Male)	37.5%	45.5%	45.5%	53.9%	43.8%
BMI (*SD*)	27.1 (6.0)	32.1 (8.2)*	28.4 (5.4)	27.4 (6.0)	28.7 (6.7)
Race					
White	75.0%	72.7%	72.7%	46.2%	69.7%
Black	12.5%	27.3%	22.7%	53.9%	24.7%
Other	12.5%	0.0%	4.6%	0.0%	5.6%
Hispanic or Latino (%)	18.8%	0.0%	13.6%	0.0%	10.1%
Smoking status					
Current	0.0%	0.0%	0.0%	100.0%	14.6%
Former	0.0%	100.0%	13.6%	0.0%	28.1%
Never	100.0%	0.0%	86.4%	0.0%	57.3%

* *p* ≤ 0.05

**** *p* ≤ 0.0001 when compared to nonsmokers.

### 2.2 Sample collection

NELF was collected by moistening the airway with approximately 100 ul of normal, sterile saline (0.9%) using a metered spray bottle, inserting strips of absorbent fibrous Leukosorb matrix (Pall Scientific) cut to fit within the nasal passage, clamping for 2 min with a padded nasal clamp, removing, placing in a microfuge tube, and freezing until analysis as described previously (Rebuli et al., 2017).

### 2.3 Sample analysis

The Leukosorb strips were eluted after thawing. The full protocol has been described elsewhere (Rebuli et al., 2017). Briefly, an elution buffer (1% bovine serum albumin [Sigma], 0.05% Triton X‐100 [Sigma], in DPBS [Sigma]) was added to the strip, then centrifuged to remove NELF from strips. NELF was then analyzed using a V‐Plex Human Cytokine 30‐Plex Kit (Meso Scale Diagnostics), according to manufacturer instructions and the minimum manufacturer recommended dilutions.

### 2.4 Statistical analysis

Raw data are available in the UNC Dataverse (https://doi.org/10.15139/S3/XHHUFB).

Mean and standard deviation (SD) were calculated for age and BMI. We used ANOVA and post hoc Dunnet's test to detect differences between groups. Relative percentages were calculated for sex, race, ethnicity, and smoking status. Any nasal immune mediators that were below the limit of detection by ELISA were set to half the minimum detected value for all analyses. We used ANOVA to detect differences in the mean levels of each nasal immune mediator (ng/ml) across the four cohorts. We reported unadjusted *p*‐values and *p*‐values adjusted with the Benjamini and Hochberg method to control the false discovery rate (FDR).

For mediators found to differ among groups by ANOVA, we determined the percent difference in average mediator concentration for each cohort compared to the healthy nonsmoking cohort. For these comparisons, the concentrations (ng/ml) were log‐transformed and we used Dunnett's test to compensate for multiple comparisons. We also ran multinomial logistic regression models to assess the association between the concentration for these mediators in the nasal fluid and the odds of being in the COPD, CRS, and smoking cohort compared to being in the healthy cohort. We ran unadjusted models and additionally adjusted models for sex. Due to lack of overlap between ages of the elderly COPD group and the other groups, we were unable to control for age statistically in this study.

In order to assess if nasal inflammatory mediators differed by anti‐inflammatory medication or respiratory symptoms, we ran sensitivity analyses within the COPD group to investigate differences in mediator concentrations by inhaled corticosteroid (ICS) use and more symptom variability (defined as ≥10 days of worse‐than‐baseline respiratory symptoms over 4 months of observation) using *t*‐tests (Alvarez‐Baumgartner et al., 2022). These sensitivity analyses were possible only in the COPD subgroup because ICS use was an exclusion criterion in the healthy and smoking groups, and data on ICS use and respiratory symptoms were only collected in the group with COPD.

In order to identify modules (clusters) of correlated mediators and their correlation with airway disease and smoking status, we conducted a weighted gene co‐expression network analysis (WGCNA) using the R4.1.1 *WGCNA* package (Langfelder & Horvath, 2008). A soft‐power threshold of 6 was chosen after determining that the scale‐free topology fit index curve flattened at 6. Modules were constructed with a merging threshold of 0.25 and minimum module size of 2, resulting in six modules. We evaluated the association of each cluster with cohort membership and display these results graphically with a heat map. Finally, we identified the specific mediators driving the clusters (hub proteins), defined as having a high cohort significance (correlation between healthy cohort membership and mediator concentration >0.2) and having strong module membership (correlation of module eigengene and gene expression profile >0.8), based on (Langfelder & Horvath, 2008).

## 3 RESULTS

Cohort demographics by disease status are presented in Table 1. The mean age of participants in this study was 42.8 ± 19.2 and 43.8% of the participants were male. The mean BMI was 28.7 ± 6.7 and was similar across the cohorts, except for the COPD cohort which was significantly higher. The COPD and CRS cohorts were significantly older than the nonsmoker cohort (mean age 71 and 41, respectively, compared to 29).

### 3.1 Individual immune mediator comparisons

Twenty‐eight cytokines and chemokines were analyzed via ANOVA across cohorts (Table S1 in online supplement). Mean levels of MCP‐1, IL‐7, IL‐10, IL‐12p70, IL‐13, and IL‐15 differed among the cohorts (ANOVA *p* < 0.05). Levels of IL‐10, IL‐12p70, IL‐13, and MCP‐1 were significantly different among cohorts after correcting for multiple comparisons (FDR‐adjusted ANOVA *p* < 0.05). For each of these six mediators, average nasal levels were highest among the healthy nonsmokers.

**FIGURE 1:**
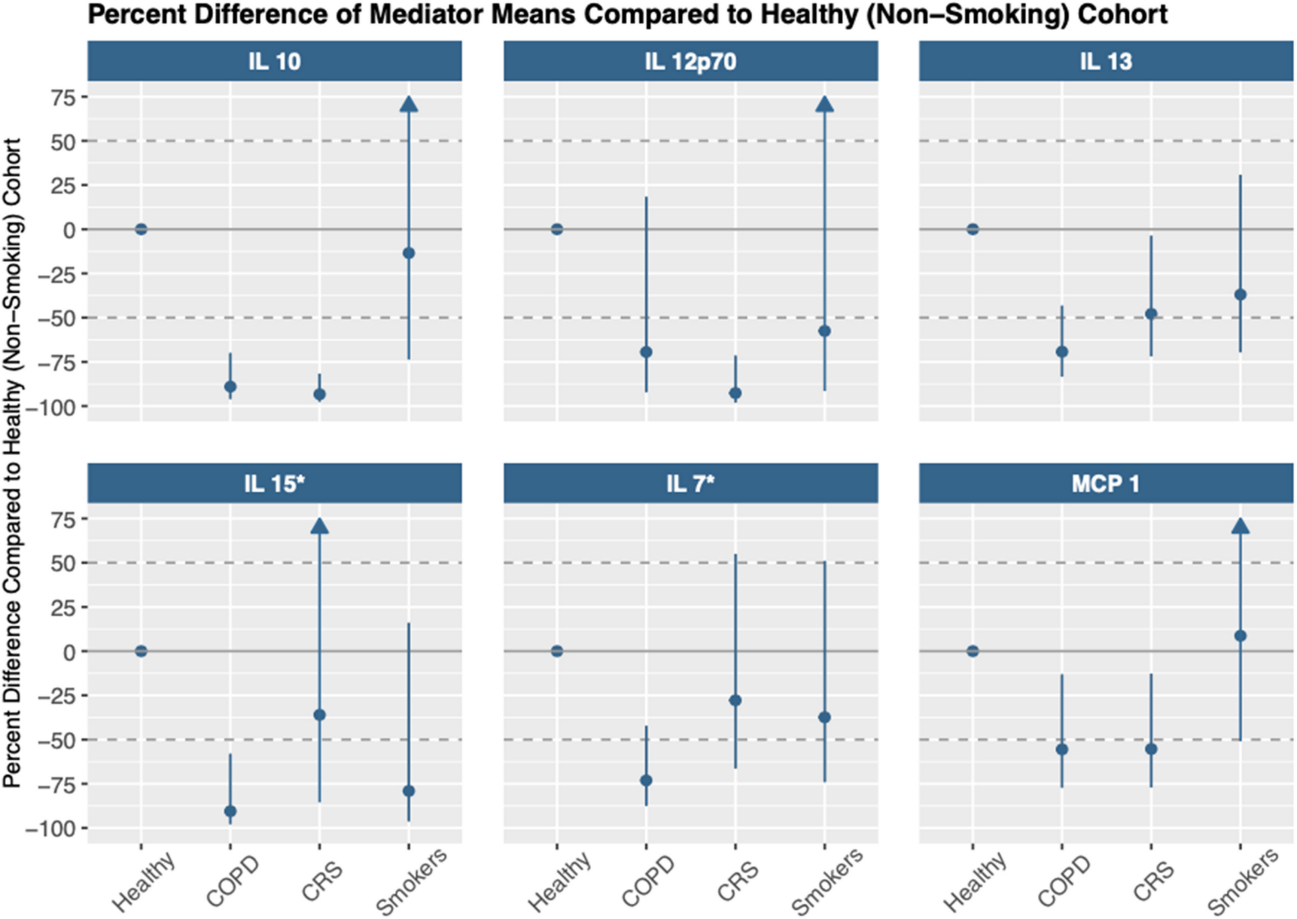
Nasal immune mediator differences across cohorts. Percent difference from healthy controls and confidence intervals are plotted for IL‐10, IL‐12p70, IL‐13, IL‐15, IL‐7, and MCP‐1. *Healthy group for these proteins only had 20 participants; Arrows indicate confidence interval extends beyond the plot axis.

Figure 1 shows the percent difference in these six mediators for each cohort compared to the healthy controls. Levels of MCP‐1, IL‐7, IL‐10, IL‐13, and IL‐15 were lower among those with COPD compared to healthy controls. Among those with CRS, levels of MCP‐1, IL‐10, IL‐12p70, and IL‐13 were lower compared to healthy controls. In the multinomial regression (Table 2, Table S1), lower nasal concentrations of IL‐10, IL‐12p70, IL‐13, and MCP‐1 were associated with higher odds of being in the COPD or CRS group compared to the healthy group. Lower IL‐15 and IL‐7 were also associated with higher odds of being in the COPD cohort. For example, an interquartile range *lower* IL‐10 concentration (contrasting a 25th percentile to the 75th percentile of the distribution) was associated with 6.4 (95% CI 1.8 to 23.2) times the odds of being in the COPD versus healthy group. The corresponding odds ratios were 6.9 (95% CI 2.1 to 22.1) for IL‐13, 4.34 (95% CI 1.5 to 12.5) for IL‐12p70, 3.6 (95% CI 1.3 to 9.4) for IL‐15, and 6.0 (95% CI 1.9 to 19.4) for IL‐7. An IQR lower level of IL‐12p70 was associated with 10.9 (95% CI 2.5 to 46.7) higher odds of CRS, while lower levels of IL‐10, IL‐13, and MCP‐1 were associated with 77.5 (95% CI 6.9 to 876.8), 4.0 (95% CI 1.5 to 10.6), and 5.0 (95% CI1.6 to 15.7) higher odds of CRS per IQR difference in concentration. Associations with smoking status were imprecise for these mediators, with no significant differences in the Figure 1 mediators detected compared to healthy controls and variable associations. Additional adjustment for sex did not affect the results.

**TABLE 2:** Association between inflammatory mediators and the odds of being in each disease group compared to the healthy group per IQR lower level of the mediator

	Cohort	Odds ratio (95% confidence interval)	
		Unadjusted	Adjusted for sex
IL 10	COPD	6.39 (1.76, 23.23)	6.36 (1.77, 22.9)
	CRS	77.53 (6.86, 876.82)	79.66 (6.79, 934.86)
	Smokers	1.02 (0.81, 1.28)	1.01 (0.8, 1.28)
IL 12p70	COPD	4.34 (1.5, 12.53)	5.23 (1.67, 16.35)
	CRS	10.87 (2.53, 46.73)	13.43 (2.98, 60.48)
	Smokers	1.49 (0.79, 2.84)	1.61 (0.75, 3.47)
IL 13	COPD	6.87 (2.13, 22.14)	7.3 (2.2, 24.26)
	CRS	3.97 (1.49, 10.59)	4.14 (1.5, 11.46)
	Smokers	2.27 (0.88, 5.89)	2.39 (0.87, 6.56)
IL 15	COPD	3.55 (1.34, 9.39)	3.61 (1.35, 9.63)
	CRS	1.53 (0.81, 2.87)	1.55 (0.82, 2.93)
	Smokers	2.48 (0.93, 6.58)	2.53 (0.94, 6.83)
MCP 1	COPD	2.19 (0.96, 5)	2.18 (0.95, 4.97)
	CRS	4.98 (1.58, 15.67)	4.96 (1.58, 15.59)
	Smokers	0.92 (0.46, 1.8)	0.9 (0.46, 1.78)
IL 7	COPD	6.01 (1.86, 19.43)	6.62 (2.01, 21.79)
	CRS	1.24 (0.66, 2.34)	1.34 (0.7, 2.59)
	Smokers	1.4 (0.64, 3.06)	1.57 (0.69, 3.55)

### 3.2 Cluster analyses

**FIGURE 2:**
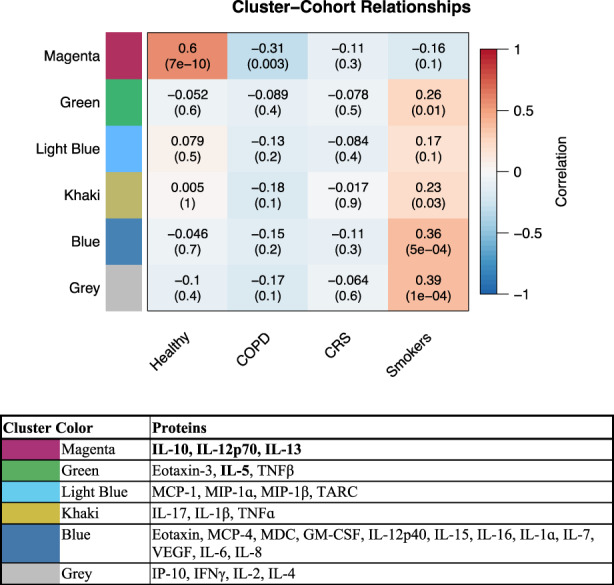
Clusters of immune mediators and correlation across disease states. WGCNA was used to identify six clusters of immune mediators that were correlated in their expression across disease state cohort. Clusters are labeled by color: Magenta, green, light blue, khaki, blue, and gray. Within the correlation matrix, *r* (*p*‐value) are shown for each cluster and cohort along with the box being colored by strength of *r* value. Proteins within each cluster are identified in the table below the matrix and hub proteins are bolded.

To describe the immune mediator profiles within each disease state and how they compare to healthy controls, we completed a weighted correlation network analysis (WGCNA), a systems biology technique to describe correlation patterns across mediators in a dataset. As cytokine and chemokine production and regulation is complex, functioning additively, synergistically or even antagonistically to regulate immune system homeostasis, we chose this systems biology approach to characterize the dynamic nature of immune mediator function. Using WGCNA, we identified six clusters of immune mediators that were correlated in their expression across disease states. Cluster identifiers and the proteins within each cluster are described in Figure 2. The magenta cluster (containing IL‐10, IL‐12p70, and IL‐13) was positively correlated with healthy status (*r* = 0.60) and negatively correlated with COPD status (*r* = −0.31). The other clusters were all primarily upregulated in the smoking cohort. Of these, the most notable contrast was the gray cluster (containing IP‐10, IFNγ, IL‐2, IL‐4) which was positively correlated with the smoking group (*r* = 0.39). Within this analysis, we also identified the primary mediators that were drivers of the clustering results or hub proteins. These included IL‐10, IL‐12p70, and IL‐13 in the magenta cluster, and IL‐5 in the green cluster. When interpreting these correlations, a positive correlation coefficient indicates that the disease state (e.g., healthy, COPD, smoking) is correlated with higher levels of the group of mediators in the cluster (taking into account the levels of all mediators within the cluster) and a negative correlation coefficient indicates the disease state is correlated with lower levels of the group of mediators. Unlike the evaluation of mean levels of individual mediators by disease state shown in Table S1 (which does not account for levels of any other mediators), the cluster correlations can be interpreted as the representative effect estimate of each cluster of mediators on the trait, taking into account the full dataset.

### 3.3 Sensitivity analysis within COPD cohort

Within the older COPD group, inflammatory mediators did not differ by more frequent symptom exacerbation (≥10 days of worse‐than‐baseline respiratory symptoms over 4 months of observation). Results suggested that two proinflammatory mediators, IL‐6 and IL‐1β, were lower among ICS users (*n* = 11) compared to those who did not use ICS (*n* = 9), albeit not statistically significant. For IL‐6, the median concentration for those who did not use ICS was 10.4 compared to 3.7 for those who used ICS (*t* = 2.0, *df* = 18, *p* = 0.06). Similarly, for IL‐1β, the median concentration for those who did not use ICS was 23.4 compared to 12.7 for those who used ICS (*t* = 1.9, *df* = 18, *p* = 0.07).

## 4 DISCUSSION

Using a novel method of sampling the nasal epithelial lining fluid in an exploratory study, we found that adults with upper and lower airway disease had lower levels of several immune mediators compared to healthy adults. Several mediators differed across the health condition‐related cohort, including IL‐10, IL‐12p70, IL‐13, IL‐15, and MCP‐1, which were generally lower among those with COPD and CRS compared to healthy adults.

Within our exploratory study, we consistently found lower levels of IL‐10 in our COPD and CRS cohorts compared to healthy adults, both in our individual mediator assessment and cluster‐based analysis. IL‐10 is a known potent anti‐inflammatory and pro‐resolving factor that has been identified as a potential indicator of overall lung health. Higher IL‐10 levels in sputum and serum have been associated with higher lung function, lower disease severity, and lower levels of inflammation in human studies. IL‐10 is produced by a variety of cellular sources including B cells, monocytes, DCs, NK cells, T cells, and epithelial cells, and based on its role in inflammatory processes, is thought to fine tune immune responses to stimuli, including pathogens and other environmental stimuli (Branchett & Lloyd, 2019; Delieva et al., 2013; Kubo et al., 2017; LeVan et al., 2018). In diseases like COPD, IL‐10 levels have been reported to be reduced in respiratory fluid and serum as disease severity increases (Delieva et al., 2013). In CRS, decrements in IL‐10 have been associated with particular phenotypes, especially CRS with nasal polyps (Liao et al., 2018). Interestingly, this phenotype is also associated with eosinophilic inflammation, potentially due to lack of anti‐inflammatory signaling balance (König et al., 2016; Oyer et al., 2013; Park et al., 2013). Additionally, IL‐10 has been shown to play a role in response to allergy, a common comorbidity of CRS and potential trigger for COPD exacerbation, by dampening Th2‐derived allergic inflammation (Jamieson et al., 2013; Jantunen et al., 2019; Marcus et al., 2019). Our study results are consistent with these observations, with lower nasal IL‐10 found to be associated with both COPD and CRS. However, it should be noted that due to small sample size we were unable to stratify our CRS cohort by disease endotype, therefore further exploration of the specificity of our findings to different endotypes is needed. Based on these results and other findings in the literature, we suggest that the suppression of critical immunomodulatory cytokines, especially those that are anti‐inflammatory such as IL‐10, could be explored in future studies as potential nasal mucosal biomarkers of respiratory disease.

We found a consistent pattern of lower levels of most immune mediators from airway samples in individuals with airway disease compared to our healthy control cohort, which differs from other studies in the field. While we found no significant differences in these respiratory mediators by disease group, others have found higher levels of Eotaxin and IFNγ in COPD and higher levels of IL‐5 and MCP‐4 in CRS, particularly those with nasal polyps, compared to healthy controls (Stevens et al., 2015). Additionally, another group found increased levels of IL‐6 in sputum samples from individuals with COPD and an inverse correlation between sputum IL‐6 and lung function (Rincon & Irvin, 2012). In this study, the lack of findings on nasal IL‐6 in COPD versus healthy controls may be due to differences in mediator production in sampling regions of the respiratory tract. This notion is supported by a prior study which found that immune mediator responses to inflammatory stimuli varied in cultures derived from nasal and bronchial cells from individuals with COPD (Comer et al., 2012). However, others have also found that inflammatory mediator production is similar in the nose and bronchi (Håkansson et al., 2013). Additionally, studies of COPD cohorts often analyze levels of circulating serum or plasma to compare with pulmonary function data and have found increased levels of mediators such as MCP‐1, IL‐6, and IL‐13 to be associated with increased disease severity and poorer lung function, further indicating that sampling region may play a role in varying results (Di Stefano et al., 2018; Lee et al., 2007). Larger studies, prospective studies across sampling location and disease status are thus needed to determine whether overlap exists in mediator production between airway compartments and whether mediator levels differ by disease status within and across sampling regions.

The overall pattern of lower immune mediator levels in our airway disease groups was unexpected, yet was confirmed in multiple healthy subject sub‐cohort analyses. The lower levels of immune mediators across our disease groups may be influenced by additional factors that cannot be controlled in this study, for example, age (due to non‐overlap of the ages of the elderly COPD group vs the younger healthy adults) or inhaled medication use. We completed a sensitivity analysis within our COPD group exploring the influence of ICS use and respiratory symptoms on nasal immune mediators and found lower IL‐6 and IL‐1β levels in our ICS group compared to those not utilizing ICS. The suppression of inflammatory mediators is not unexpected with corticosteroid use and has been well documented (Barnes, 2010; Tanaka et al., 2014).

In smokers, we found that none of the individual immune mediators differed compared to healthy adults. There were several clusters of mediators that were positively correlated with smoking status, although the strength of the cluster correlations was modest. These include a cluster with IP‐10, IFNγ, IL‐2, IL‐4 (*r* = 0.39, gray, Figure 2), and a cluster with IL‐5, Eotaxin, and TNFβ (*r* = 0.26, green, Figure 2). Differences in clusters of mediators may identify patterns among immune mediators with interacting correlated functions that are not evident when evaluating individual mediators alone, a finding that has previously been reported in other airway biomarker studies of smokers (Payton et al., 2022). These clusters have some biological plausibility, as increases in included mediators have been reported with cigarette smoke exposure in multiple studies. For example, increases in IP‐10 were observed with cigarette smoke exposure in both rodent and respiratory cell culture models (Botelho et al., 2010; Rajendrasozhan et al., 2010; Wang et al., 2012). Similarly, IL‐4 has been reported to be increased in cigarette smokers (Byron et al., 1994; Daloee et al., 2017) and IL‐5 levels are increased in mouse BAL following cigarette smoke exposure (Obot et al., 2004). Eotaxin was also found to be elevated in BAL of current cigarette smokers compared to nonsmoking controls in another study (Miller et al., 2007). Overall, trends in cluster association with cigarette smoking were observed that are mostly consistent with findings in the literature. Individual mediators were not different when compared to healthy nonsmokers, which does deviate from the literature, but could be explained by our relatively small sample size or sampling region of choice.

There are several limitations of this study that should be addressed in future work in order to confirm the biological significance of our findings. Our study included relatively small sample sizes, which limited power to detect differences. We were unable to adjust for multiple factors due to enrollment criteria in the individual groups secondary to the retrospective design of the study, including age, as there was no overlap between the COPD group and the other groups (the COPD group was significantly older), and medication use, which may have affected levels of these mediators in the nose. To partially address the potential influence of confounders, a sensitivity analysis within the COPD group was completed. The comparison of ICS in the COPD group indicated that there may be lower levels of certain proinflammatory mediators for those who use ICS, but data on ICS use was limited to the COPD group. We also included 28 mediators in our analyses, thus increasing the potential type I error rate, however addressed this limitation by calculating FDR‐adjusted p‐values.

Overall, our study demonstrates potential differences in the patterns of nasal immune mediators in individuals with a variety of respiratory diseases and risk factors. Five individual mediators were different among the cohorts (MCP‐1, IL‐7, IL‐10, IL‐13, and IL‐15) with lower concentrations in the groups with respiratory disease, particularly COPD and CRS. Similar patterns were also found in our cluster‐based analyses, with clustered groups of correlated mediators showing lower expression levels in the upper and lower respiratory disease groups. Our findings suggest that certain immune mediators, such as IL‐10, may have utility in distinguishing healthy adults from those with lung disease, but these results will need to be confirmed in a larger prospective study designed to test this hypothesis. Our findings also suggest tobacco smoke affects the nasal immune environment when sampled by nasosorption. As chronic respiratory conditions can be caused by a variety of toxicant exposures, there is a critical need to identify early biomarkers of these conditions to promote early detection and appropriate treatment or mitigation, based on exposures. This exploratory study further suggests that the nasal passage may be an ideal target for biomarker detection. It has already been demonstrated that serum biomarkers are often not the best representative source for information on respiratory immune status; rather, airway samples, even from sources such as the nose, are better correlates for lower airway than circulating blood (Payton et al., 2022). In the future, nasosorption may be a feasible method to evaluate respiratory biomarkers of disease or exposure in the clinical environment with further validation.

## AUTHOR CONTRIBUTIONS

MER, IJ, and MBR conceptualized this work. All authors participated in the acquisition (MER, WYS, AJK, CSE, MA, IJ, MBR), analysis (ASL, LN, UAT, WYS), or interpretation of data (MER, ASL, LN, UAT, WYS, IJ, MBR) for the work and all authors drafted or revised the manuscript critically for intellectual content. All authors provided final approval of the manuscript to be published and agree to be accountable for all aspects of the work in ensuring that questions related to the integrity of any part of the work are appropriately investigated and resolved.

## FUNDING INFORMATION

This research was supported by the Flight Attendant Medical Research Institute (CIA_160016), National Institute of Environmental Health Sciences (T32ES007126, K23ES026204, P30ES000002 and R01ES031252), National Heart, Lung, and Blood Institute (P50HL12010004), and the Leon and Bertha Golberg Award. Research reported in this publication was in part supported by the U.S. National Institutes of Health and the U.S. Food and Drug Administration Center for Tobacco Products. The content is solely the responsibility of the authors and does not necessarily represent the official views of the National Institutes of Health or the U.S. Food and Drug Administration.

## CONFLICT OF INTEREST

The authors report no known conflicts of interest.

## Supporting information


Supplementary Table S1.
