# Supplementary material for: Nasal biomarkers of immune function differ based on smoking and respiratory disease status
Source: Physiol Rep. 2023 Feb 13;11(3):e15528. doi: 10.14814/phy2.15528 (PMC9925276; doi:10.14814/phy2.15528)
Supplement: Supplementary file 1 — Supplementary Table S1. [file PHY2-11-e15528-s001.docx]

Supplement eTable 1: Median (IQR) mediator levels and ANOVA comparing mean mediator levels across groups

| Mediator | Healthy  (Non-Smokers) N=32* | COPD N=22 | CRS N=22 | Smokers N=13 | Overall  N=89 | ANOVA  p-value Across Cohorts | FDR adjusted ANOVA  p-value Across Cohorts |
| --- | --- | --- | --- | --- | --- | --- | --- |
| Eotaxin | 146.09  (59.83, 247.33) | 85.45  (52.53, 187.62) | 100.25  (59.13, 230.23) | 132.2  (62.39, 278.92) | 112.98 (55.69, 248.23) | 0.59 | 0.72 |
| Eotaxin-3 | 18.02  (8.40, 73.15) | 4.74  (1.14, 14.23) | 22.36  (10.40, 74.05) | 18.74  (5.64, 99.86) | 15.07 (5.59, 45.07) | 0.46 | 0.70 |
| GM-CSF | 0.84  (0.58, 1.90)* | 0.48  (0.25, 1.06) | 0.61  (0.22, 0.88) | 0.53  (0.28, 1.28) | 0.65 (0.38, 1.27) | 0.34 | 0.67 |
| IFNγ | 7.03  (2.87, 11.53) | 3.62  (1.74, 12.99) | 1.14  (0.17, 6.52) | 4.86  (0.32, 8.93) | 4.9 (1.28, 10.39) | 0.24 | 0.57 |
| **IL-10** | 4.26  (2.49, 11.43) | 0.59  (0.15, 2.89) | 0.32  (0.15, 1.28) | 2.75  (2.04, 6.84) | 1.75 (0.53, 4.69) | **0.01** | **0.05** |
| IL-12p40 | 13.99  (6.93, 21.48)* | 5.60  (1.97, 14.14) | 7.11  (3.47, 17.05) | 6.61  (2.22, 10.39) | 8.76 (3.18, 17.05) | 0.49 | 0.70 |
| **IL-12p70** | 2.30  (0.86, 4.59) | 0.50  (0.21, 1.71) | 0.18  (0.01, 1.04) | 1.66  (1.02, 3.51) | 1.04 (0.2, 2.72) | **0.00** | **0.01** |
| **IL-13** | 39.11  (23.85, 78.16) | 14.43  (5.35, 31.87) | 18.79  (10.17, 39.07) | 31.04  (10.75, 48.23) | 26.94 (12.01, 50.47) | **0.00** | **0.01** |
| **IL-15** | 6.00  (2.60, 11.45)* | 3.28  (0.01, 5.01) | 3.56  (2.01, 5.84) | 1.13  (0.74, 1.59) | 3.39 (0.91, 6.74) | **0.02** | **0.09** |
| IL-16 | 870.59  (407.36, 1801.80)* | 425.76  (23.31, 1972.55) | 551.44  (234.48, 1396.67) | 220.67  (69.1, 980.64) | 542.4 (180.27, 1516.74) | 0.84 | 0.90 |
| IL-17 | 5.02  (1.99, 11.26)* | 0.92  (0.28, 7.06) | 1.47  (0.78, 2.02) | 1.41  (0.56, 3.65) | 1.55 (0.65, 5.41) | 0.51 | 0.70 |
| IL-1α | 251.24  (181.73, 440.78)* | 150.7  (89.04, 231.34) | 198.64  (103.12, 314.91) | 143.25  (99.27, 425.52) | 197.54 (113.68, 339.77) | 0.69 | 0.77 |
| IL-1β | 73.31  (29.64, 120.66) | 16.19  (8.20, 112.97) | 42.08  (17.51, 67.65) | 24.51  (12.84, 90.17) | 36.31 (13.9, 105.74) | 0.96 | 0.96 |
| IL-2 | 2.02  (0.01, 4.09)* | 0.99  (0.42, 5.79) | 0.45  (0.17, 3.08) | 1.70  (0.01, 5.10) | 0.95 (0.15, 4.13) | 0.09 | 0.32 |
| IL-4 | 0.69  (0.04, 2.15) | 0.18  (0.12, 0.78) | 0.05  (0.01, 0.21) | 0.99  (0.00, 2.26) | 0.25 (0.04, 0.99) | 0.07 | 0.29 |
| IL-5 | 0.12  (0.01, 1.89)* | 0.09  (0.00, 0.46) | 1.60  (0.14, 6.42) | 0.01  (0.01, 0.14) | 0.13 (0.01, 1.85) | 0.38 | 0.68 |
| IL-6 | 13.26  (7.41, 25.14) | 7.35  (2.14, 35.78) | 5.81  (2.46, 35.75) | 7.64  (1.65, 18.10) | 10.29 (3.6, 26.54) | 0.42 | 0.70 |
| **IL**-**7** | 83.89  (49.93, 111.30)* | 41.43  (7.78, 61.33) | 66.3  (25.69, 112.03) | 48.2  (20.09, 118.47) | 52.25 (23.43, 90.01) | **0.01** | **0.08** |
| IL-8 | 27888.42  (9181.96, 44300.80) | 4248.67  (1002.18, 16268.30) | 3964.24  (2824.64, 10553.37) | 13122.85  (8383.89, 33118.01) | 9760.97 (3339.43, 31764.26) | 0.28 | 0.58 |
| IP-10 | 1985.07  (454.82, 6442.56) | 1777  (304.43, 3586.36) | 1378.11  (455.31, 7739.34) | 587.99  (281.40, 4309.29) | 1451.12 (354.96, 4490.89) | 0.14 | 0.37 |
| **MCP**-**1** | 297.97  (207.07, 555.88) | 211.81  (72.58, 494.52) | 169.19  (75.52, 214.42) | 435.8  (151.29, 604.87) | 230.54 (126.18, 491.38) | **0.01** | **0.05** |
| MCP-4 | 18.52  (12.43, 29.85)* | 15.87  (5.91, 26.53) | 15.37  (9.56, 66.48) | 18.99  (12.57, 31.33) | 17.35 (10.13, 29.97) | 0.28 | 0.58 |
| MDC | 85.98  (45.40, 111.67)* | 99.29  (30.87, 145.23) | 86.8  (43.16, 121.12) | 64.69  (55.84, 81.54) | 85.68 (44.15, 121.12) | 0.91 | 0.94 |
| MIP-1α | 55.96  (26.84, 72.28) | 29.75  (17.88, 50.85) | 37.07  (19.04, 65.84) | 28.56  (7.92, 45.28) | 35.62 (20.68, 70.23) | 0.65 | 0.76 |
| MIP-1β | 95.08  (53.85, 153.33) | 35.06  (14.66, 117.84) | 66.74  (29.42, 114.23) | 50.75  (18.04, 74.62) | 74.07 (29.42, 127.14) | 0.53 | 0.70 |
| TARC | 24.58  (15.17, 37.53) | 21.19  (11.30, 31.69) | 21.47  (13.20, 36.41) | 22.37  (10.72, 29.66) | 22.37 (12.29, 33.19) | 0.45 | 0.70 |
| TNFα | 5.74  (2.66, 9.97) | 2.47  (0.65, 8.19) | 3.37  (2.09, 16.36) | 2.28  (0.84, 4.58) | 3.92 (1.18, 8.83) | 0.12 | 0.37 |
| TNFβ | 0.15  (0.00, 0.66)* | 0.00  (0.00, 0.00) | 0.31  (0.11, 0.54) | 0.10  (0.00, 0.28) | 0.07 (0, 0.36) | 0.59 | 0.72 |
| VEGF | 2757.52  (1558.60, 3288.70)* | 1248.24  (366.23, 2816.69) | 2256.81  (1342.86, 3743.93) | 2206.15  (1414.15, 3105.46) | 2206.15 (1246.98, 3137.19) | 0.13 | 0.37 |
| *For healthy group, only 20 samples analyzed for proteins marked with asterisk  Mediators with ANOVA significant at p=.05 in bold  Medians reported as ng per mL | | | | | | | |
